# Anthropogenic gadolinium in the Tone River (Japan): an update showing a 7.7-fold increase from 1996 to 2020

**DOI:** 10.1186/s41747-024-00460-2

**Published:** 2024-05-24

**Authors:** Soma Kumasaka, A. Adhipatria P. Kartamihardja, Yuka Kumasaka, Satomi Kameo, Hiroshi Koyama, Yoshito Tsushima

**Affiliations:** 1 Department of Diagnostic Radiology and Nuclear Medicine, Gunma University Graduate School of Medicine, 3-39-22 Showa-machi, Maebashi, Gunma 371-8511 Japan; 2 Radiological Sciences, School of Medicine, University of Nottingham, Nottingham, NG7 2UH United Kingdom; 3 Department of Nuclear Medicine and Molecular Imaging, Universitas Padjadjaran, Jl. Raya Bandung Sumedang KM.21, Hegarmanah, Kabupaten Sumedang, Jatinangor, Jawa Barat 45363 Indonesia; 4 Department of Nutrition, Koshien University, 10-1 Momijigaoka, Takarazuka, Hyogo 665-0006 Japan; 5 Department of Public Health, Gunma University Graduate School of Medicine, 3-39-22 Showa-machi, Maebashi, Gunma 371-8511 Japan; 6 Division of Internal Medicine, Gunma Rehabilitation Hospital, 2136 Kamisawatari, Nakanojo, Agatsuma District, Gunma, 377-0541 Japan

**Keywords:** Anthropogenic effects, Environmental monitoring, Gadolinium, Magnetic resonance imaging, Water

## Abstract

**Background:**

Anthropogenic gadolinium (Gd), originating from Gd-based contrast agents (GBCAs) used in magnetic resonance imaging (MRI), is widely identified in the aquatic environment with concerns about toxicity and accumulation. We aimed to present new data on anthropogenic Gd in the Tone River, which has the largest drainage area in Japan, and then to compare the current data with those obtained in 1996.

**Methods:**

The water samples were collected on August 9−10, 2020, at 15 different locations of the Tone River in Japan. The concentrations of the rare earth elements (REEs) were measured by inductively coupled plasma-mass spectrometry and normalized to Post-Archean Australian Shale to construct shale-normalized REE patterns. The degree of Gd-anomaly was defined as the percentage of anthropogenic Gd to the geogenic background and used to compare the water samples from different locations. Pearson’s correlation coefficients were calculated.

**Results:**

All the samples displayed positive Gd anomalies. The Gd-anomaly ranged from 121 to 6,545% and displayed a repeating decrease-and-increase trend. The Gd-anomaly showed strong positive correlations to the number of hospitals (*r* = 0.88; *p* < 0.001) and their MRI units (*r* = 0.89; *p* < 0.001).

**Conclusions:**

Our study revealed notable anomalies of Gd concentrations in river water in Japan, with strong positive correlations to the number of major hospitals and their MRI units. Compared with the previous report in 2000, the Gd-anomaly in Tone River increased from 851% (sampled in 1996) to 6,545%, *i.e.,* 7.7 times, reflecting the increased use of GBCAs in hospitals.

**Relevance statement:**

Notable Gd concentration anomalies in river water in Japan were observed. This result underlines the importance of more extensive research on anthropogenic gadolinium, and investigations of risks to human health as well as the development of effective removal technologies may be necessary.

**Key points:**

• All water samples from Tone River displayed positive Gd anomalies.

• The Gd anomalies increased to 7.7 times higher over the past 24 years.

• Correlations between Gd values and the number of hospitals and MRI units were observed.

**Graphical Abstract:**

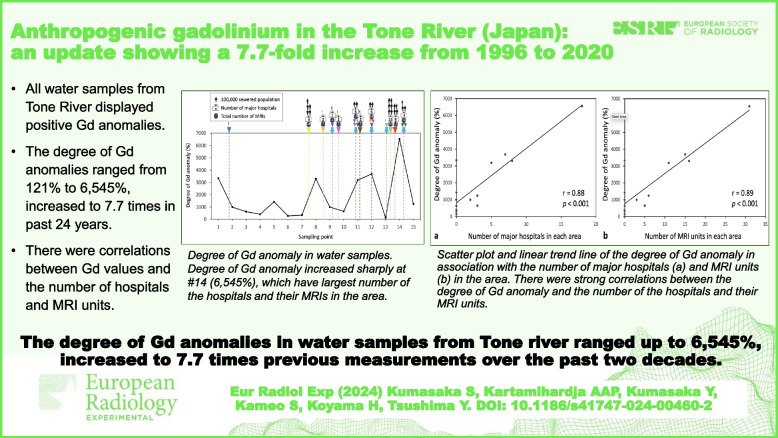

## Background

Gadolinium (Gd) is one of the rare earth elements (REEs). It has been widely used as an intravenous contrast agent for magnetic resonance imaging (MRI) examinations in clinical practice to improve diagnostic sensitivity and accuracy. To avoid toxicity, Gd is used in a chelated form as a Gd-based contrast agent (GBCA). Gd keeps its paramagnetic characteristics in this form, while its toxicity is strongly reduced [1]. After their use, these compounds are removed by the renal and hepatic systems and rapidly excreted in the urine and feces [2]. Therefore, GBCAs were thought to be safe until recently.

However, for more than 20 years, there have been several concerns regarding the safety of GBCAs. In 1997, nephrogenic systemic fibrosis was first identified among renal transplant recipients [3]. Occurring in patients with severe renal impairment, it is a systemic fibrosing disorder caused by dechelated, free Gd. It is characterized by skin and subcutaneous thickening and can lead to multiple organ failure and death [4, 5]. This new finding has led to the reconsideration of the safety of GBCAs. Furthermore, Gd deposition in brain tissue detected by MRI after administration of GBCA was first reported in 2014 [6]. It has also been reported that Gd deposition in the brain may not be ruled out even if Gd is not detected in the brain on MRI [7]. On the other hand, unlike nephrogenic systemic fibrosis, there are increasing reports that Gd deposition in the brain is less likely to be clinically symptomatic, especially if macrocyclic GBCAs were used [8–10]. In addition, a novel agent is being developed that can achieve the same diagnostic effect with half the dose of conventional GBCAs due to the higher r1 relaxivity [11]. It has also been reported that deposited Gd in the body after the use of macrocyclic GBCAs is eliminated more rapidly than that of linear GBCAs [10, 12]. However, heavy metal deposition in the body is likely undesirable and the effects of oral intake are still unknown. In recent studies with pregnant mice, Gd deposition in the pups’ brains and their behavioral changes after injection of GBCA was reported [13, 14].

Conversely, after using GBCAs, they are discharged into the environment due to the absence of a specific removal process, even in wastewater treatment plants (WWTPs). As a result, the anthropogenic Gd is commonly identified in the aquatic environment [15–20]. Furthermore, anthropogenic Gd has been detected not only in surface water such as rivers, but also in tap water. It has also been detected in carbonated drinks from well-known fast-food chains in Germany, as many fast-food restaurants serve soft drinks made from concentrated syrup diluted with tap water [21]. These anthropogenic Gd are therefore ingested orally into the human body on a daily basis. The release of GBCAs from WWTPs from hospital waste treatment is now commonly recognized as the origin of these anomalies [22]. Although some attempts have been undertaken to mitigate the potential impacts of anthropogenic Gd on the environment by collecting patients’ urine to recycle that Gd, and a proposal has been made to retrieve Gd at WWTPs, there is no evidence or technology for the removal and recycling of Gd to date [23, 24].

Currently, Japan has the highest number of MRI scanners per capita globally, followed by the United States [25]. However, the impacts of the anthropogenic Gd on environments have been rarely studied in Japan. This study presents new data on the anthropogenic Gd in a Japanese river (Tone River) and attempts to assess the impact of anthropogenic effects through comparison with a past report.

## Methods

### Study area and sampling

The Tone River runs through suburban areas gathering tributaries of the Kanto region in Japan and pours into the Pacific Ocean. It is the second-longest river (322 km) and has the largest drainage area (16,840 km^2^) in Japan.

**Fig. 1:**
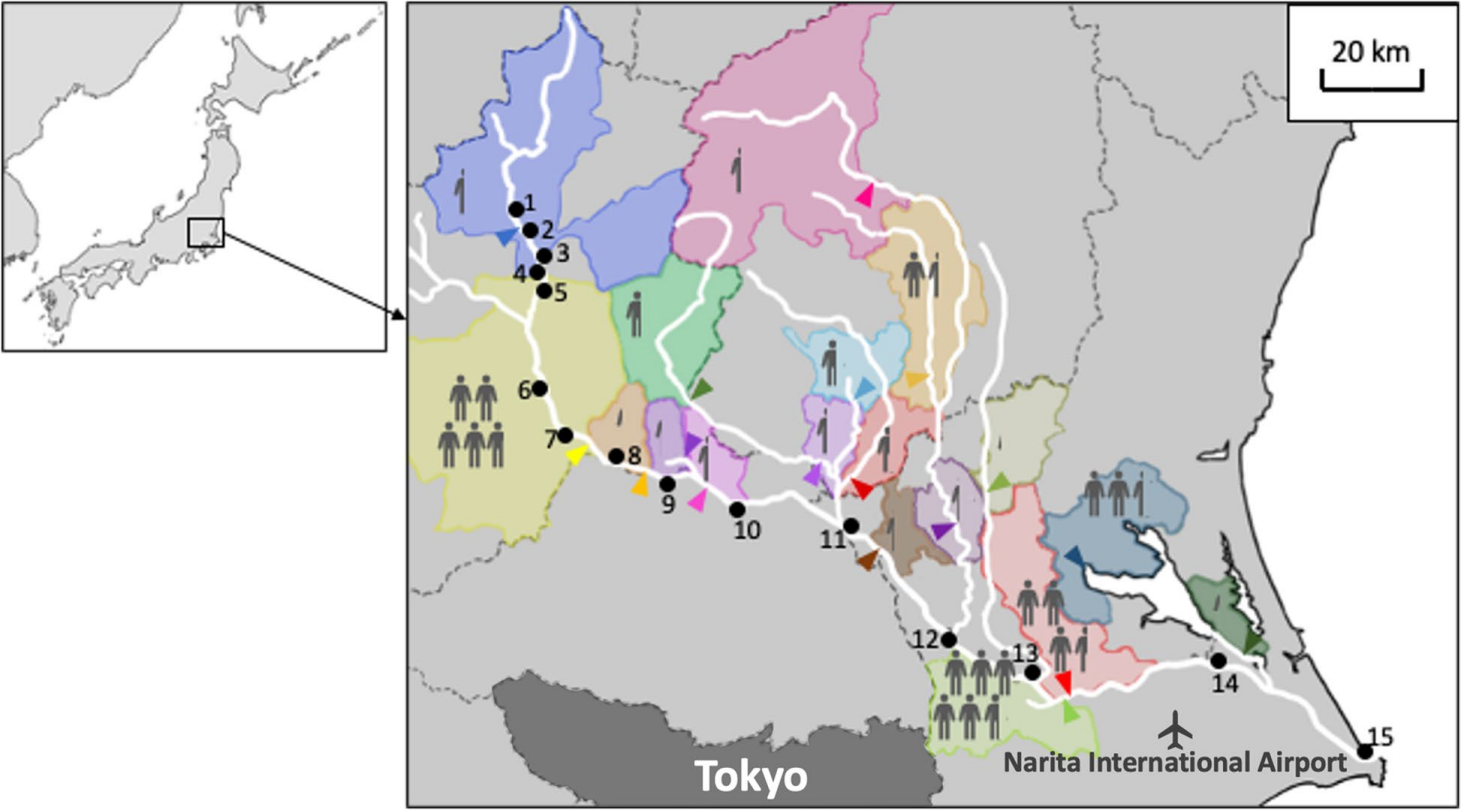
Sampling spots in the Tone River. The number corresponds to the sample code in Table 2 and Figs. 3 and 4. The colored area indicates each wastewater treatment area, and the arrowhead indicates the locations of each wastewater treatment plant output. Each human symbol represents approximately 100,000 sewered population in each area

River waters were sampled at 15 different spots in August 2020; #1–#10 were sampled on August 9, and #11–#15 were sampled on August 10. These sample points were placed approximately every 5 km in the upstream areas to increase reliability as a baseline. In the mid- to downstream areas, they were placed preferably between WWTP outputs or the confluence of tributaries to examine the effects of effluent and confluence dilution. The sampling locations in the present study are displayed in Fig. 1. For each sample, river water was taken at 10 cm deep from the water surface and 100 cm from the riverbank. Samples were collected in acid-cleaned polypropylene bottles (10 mL).

**Fig. 2:**
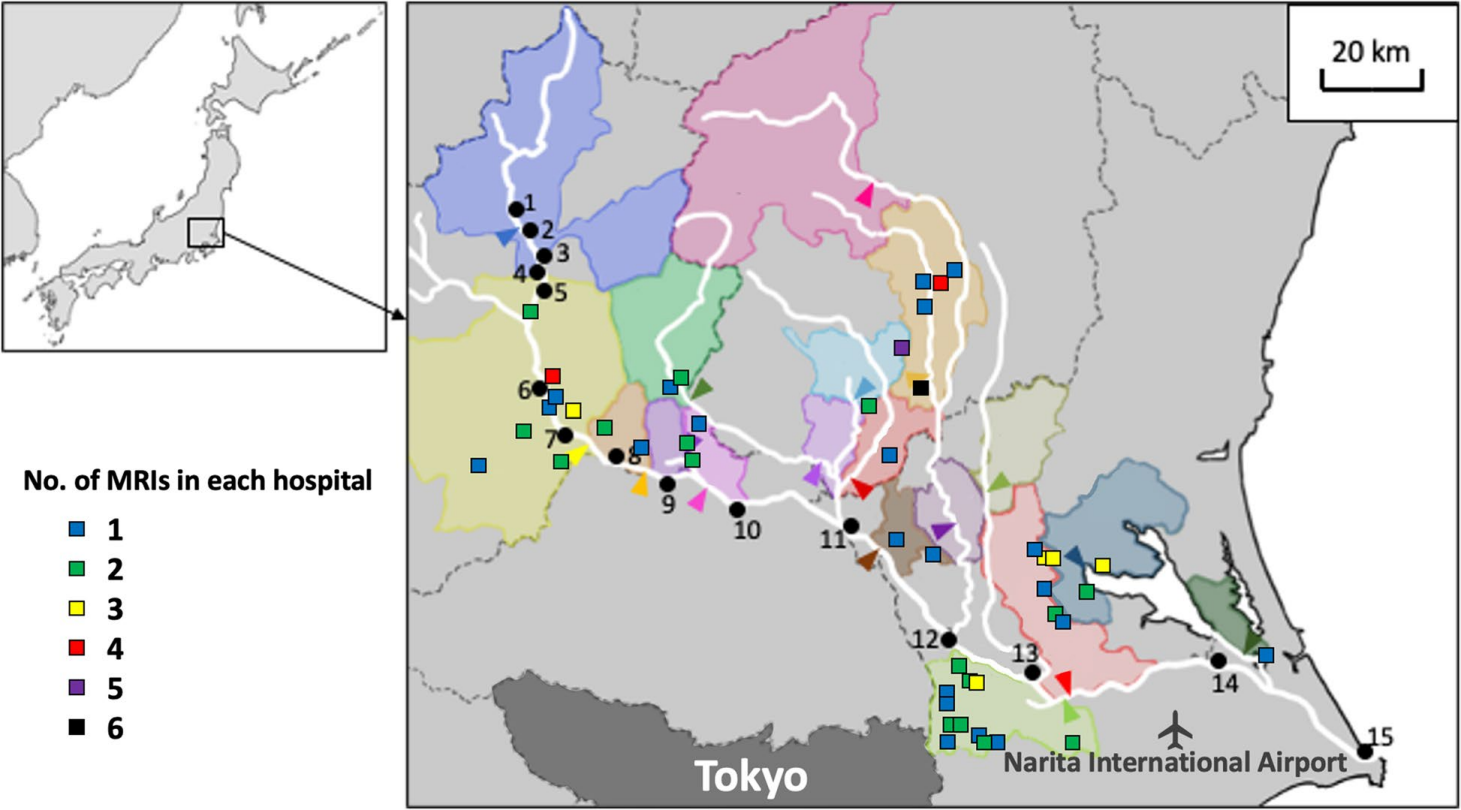
The location of major hospitals in each wastewater treatment area. The number corresponds to the sample code in Table 2 and Figs. 3 and 4. The colored area indicates each wastewater treatment area while the arrowhead indicates the locations of each WWTP output. Each square symbol represents a general hospital with more than 300 beds. The color of the square symbols represents the number of magnetic resonance imaging units owned by each hospital

The number of general hospitals with more than 300 beds in each wastewater treatment area and the number of MRI units they have were also recorded based on statistics published by the Ministry of Health, Labour and Welfare [26]. The locations of these hospitals are displayed in Fig. 2.

### REEs analysis

The reagents and solvents used in this study were all of analytical grade, ensuring the highest level of purity and accuracy. The glassware and analytical tubes underwent a 24-h suspension in a bath containing a 5% nitric acid solution and were then subjected to a thorough washing process using Ultra-purified Milli-Q water (Fujifilm Wako Pure Chemical Industries, Ltd., Osaka, Japan). A 0.22-μm filter was used to filter all samples for optimal results. Ultra-purified Milli-Q water containing 3% nitric acid was used as a blank solution, while Inductively Coupled Plasma Multielement Standard (Supelco Inc., Tokyo, Japan) solution containing 10 parts per billion (ppb) ^114^Indium (In) was used as standard solutions. A 10-mL sample of river water, comprising 3% nitric acid and 10 ppb of ^114^In, was prepared for REEs quantification. All samples were subjected to inductively coupled plasma mass spectrometry using the ELAN DRC II system (PerkinElmer, Inc.; Waltham, MA, USA). Prior to conducting instrumental analysis, a certified reference material (Elan 6100 DRC, Lot No 14-4GSL1, PerkinElmer, Inc., Waltham, MA, USA) containing representative analytes across the entire mass range was used to enhance the instrument’s sensitivity. Subsequently, an internal standard with known REE concentrations was used to confirm the measurement accuracy. The final concentration for each sample was obtained by calculating the REE signal intensity in relation to the ^114^In signal intensity. A linear regression analysis was performed to validate the measurement of REE concentration up to 4 ng/L (*R* = 0.99), using different concentrations of the REE standard. The limit of detection (LoD) and the limit of quantification (LoQ) of each measured element were measured accordingly [27] and are described in Table 1.

**Table 1:** LoD/LoQ of the measured rare earth elements

	Signal LoQ/LoD	Calculated LoQ/LoD
Ce	23.73	0.0016
Dy	8.14	0.000005
Er	8.77	-0.0008
Eu	15.15	-0.000923
Gd	30.55	0.0052
Lu	15.04	0.0022
Nd	13.73	0.00013
Pr	21.69	-0.00129
Sm	11.84	0.0051
Sc	22,881.4	-0.5628
Tb	21.6	0.0016
Yb	14.11	0.006
Y	51.68	-0.0085

*LoD* Limit of detection, *LoD* Limit of quantification

### Calculation of anthropogenic Gd concentrations

The geogenic background of Gd (from now on “geogenic Gd”) also needs to be known to determine the concentration of anthropogenic Gd. The geogenic Gd can be quantified by interpolation with neighboring elements. Therefore, all REE concentrations measured by inductively coupled plasma mass spectroscopy were normalized to Post-Archean Australian Shale (which is considered continent-wide, average compositional representatives of the post-Archean upper continental crust and widely used to distinguish background REE from anthropogenic REE) to construct shale-normalized REE patterns [28]. Geogenic Gd was then calculated using the following equation [29]:

1 $${{\text{Gd}}}_{{\text{geo}}}={{\text{Sm}}}_{{\text{SN}}}\times 0.33+{{\text{Tb}}}_{{\text{SN}}}\times 0.67$$

where Gd_geo_ is geogenic Gd, Sm_SN_ is shale-normalized samarium, and Tb_SN_ is shale-normalized terbium.

Due to the common anomaly of europium, samarium was used for the calculation as previously described [30].

The degree of the Gd anomaly needs to be objectively evaluated to compare the anthropogenic Gd among the water samples from different locations. However, the Gd concentrations are influenced by rainfall or REEs in the rocks and soils of the river basin. Therefore, the degree of Gd anomaly was defined as the percentages of the anthropogenic Gd to the geogenic Gd (Gd-anomaly) using the following Eq. 2 [18]:

2 $${{\text{Gd}}}_{{\text{anomaly}}}=\frac{{{\text{Gd}}}_{{\text{SN}}}-{{\text{Gd}}}_{{\text{geo}}}}{{{\text{Gd}}}_{{\text{geo}}}}\times 100 (\%)$$

### Statistical analysis

Pearson’s correlation coefficients were calculated to describe associations between Gd-anomaly and the total number of major hospitals and their MRI units in wastewater treatment areas between each sampling point. A *p*-value of < 0.05 was considered significant.

## Results

**Fig. 3:**
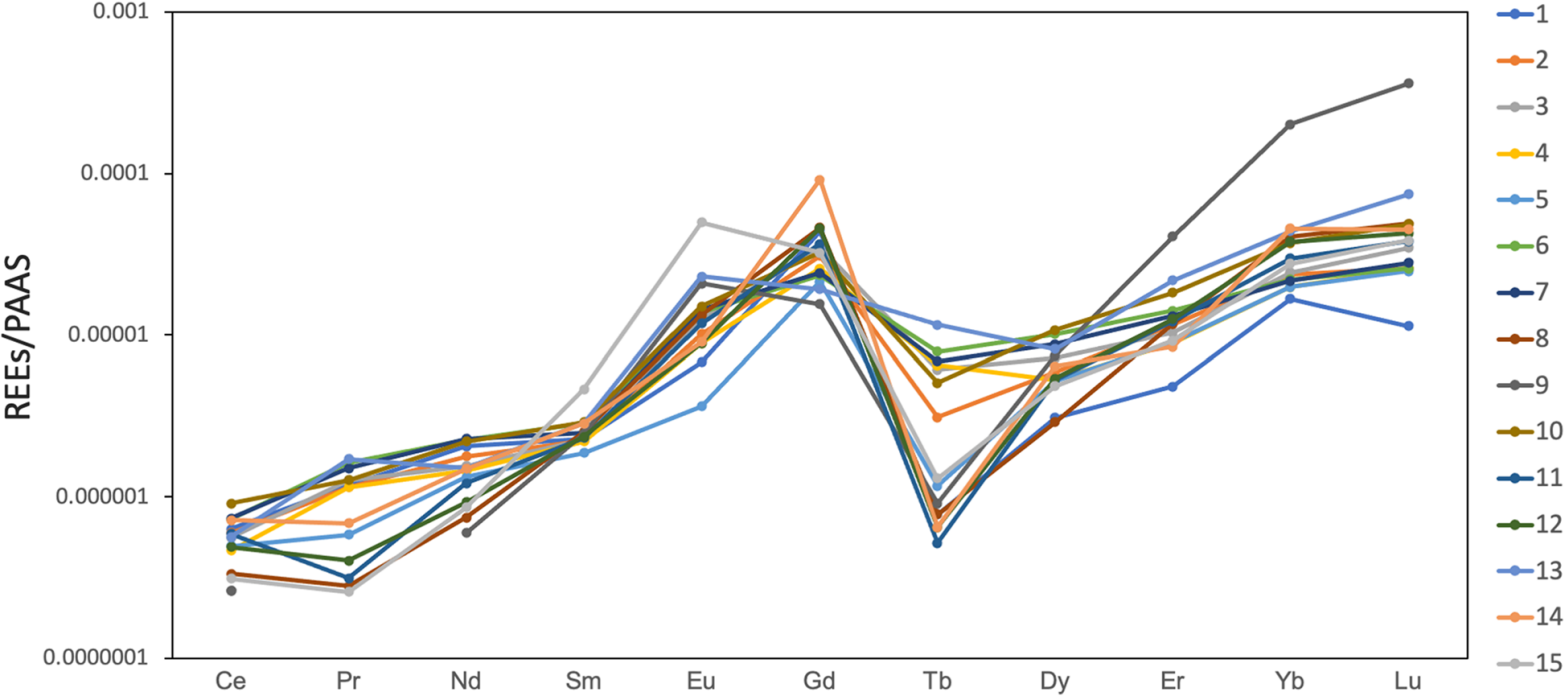
Shale-normalized rare earth elements distribution in the Tone River. All the samples showed positive Gd anomalies by interpolations with neighboring elements. Note that Sm and Tb were used for evaluation due to the common anomaly of Eu

**Fig. 4:**
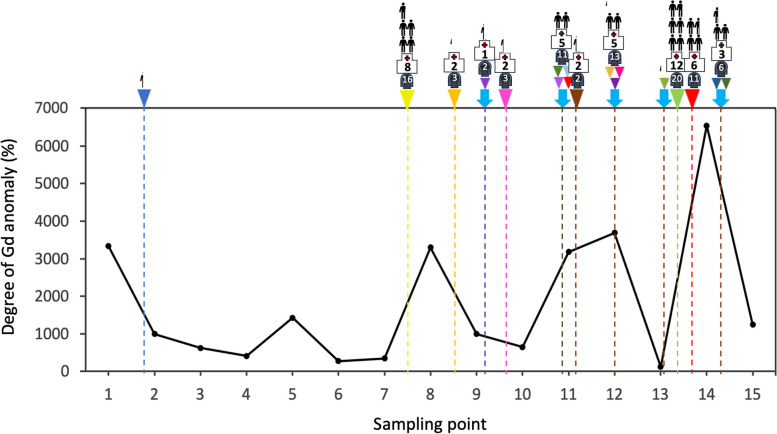
Degree of Gd anomaly in water samples from the Tone River. The arrowheads indicate the locations of each WWTP output, and light blue arrows indicate each confluence of tributaries. Each human symbol represents approximately 100,000 sewered population in each area. The numbers in the hospital symbols indicate the number of major hospitals in each area, while the numbers in the MRI symbols indicate the total number of magnetic resonance imaging units those hospitals have

The measured REE concentrations in the 15 sampling points are presented in Table 2. All the samples showed positive Gd anomalies (Fig. 3). The Gd-anomaly ranged from 121 to 6,545% (Fig. 4) and revealed a repeating decrease-and-increase trend, *i.e.*, a zigzag distribution. The largest sewered population in the area is at #14; the sample here displayed the highest Gd-anomaly (6,545%).

**Table 2:** The dissolved concentrations of rare earth elements (ng/L) in the Tone River water samples

Location	Ce	Pr	Nd	Sm	Eu	Gd	Tb	Dy	Er	Yb	Lu
1	50.7	10.5	65.6	12.8	7.5	206.0	0.6	13.6	14.0	47.0	4.9
2	47.9	10.2	57.0	12.5	11.2	145.3	2.4	25.9	33.7	66.4	11.1
3	44.8	11.3	49.8	12.6	13.7	164.6	4.7	31.7	30.0	68.2	14.9
4	37.2	10.2	46.4	12.3	9.8	121.1	5.0	23.1	25.9	55.5	11.0
5	39.6	5.2	42.4	10.5	4.0	100.4	0.9	22.8	26.1	55.7	10.7
6	58.3	14.4	71.7	16.0	15.3	110.5	6.1	44.7	40.9	61.6	11.1
7	59.1	13.4	73.3	14.0	15.8	113.7	5.3	38.6	38.0	60.5	12.0
8	26.8	2.5	23.8	14.2	14.6	217.1	0.6	12.8	34.1	113.4	21.0
9	21.1	< 0.1	19.2	13.8	22.9	73.2	0.7	32.9	118.0	562.6	155.0
10	72.9	11.3	70.7	16.3	16.6	152.1	3.9	47.2	53.1	103.7	20.9
11	47.2	2.8	38.8	13.0	13.0	171.9	0.4	23.2	35.4	83.7	16.4
12	39.2	3.6	29.8	13.2	9.8	216.0	0.5	23.7	36.5	105.6	18.3
13	45.4	15.3	48.1	15.9	25.4	90.1	8.9	36.2	63.3	122.7	31.9
14	57.6	6.1	47.8	15.9	10.0	428.5	0.5	28.3	24.6	127.9	19.4
15	25.0	2.3	27.6	26.0	54.9	151.6	1.0	21.3	26.8	77.2	16.6

**Fig. 5:**
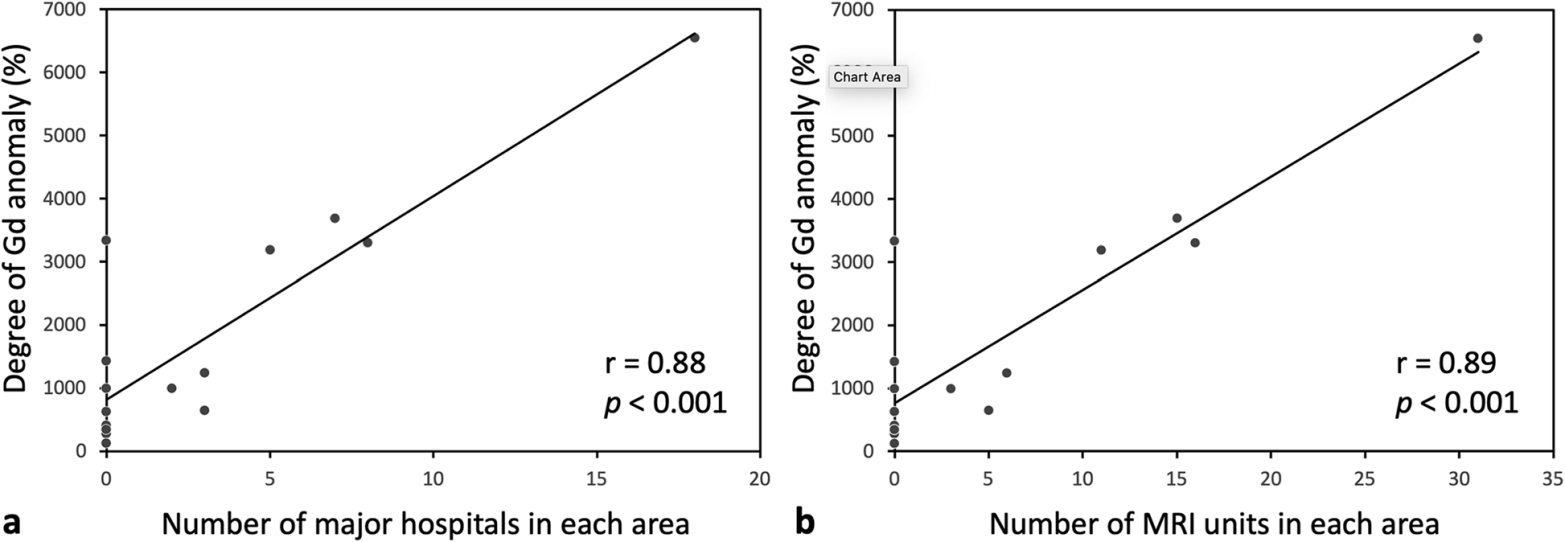
Scatter plot and linear trend line of the degree of Gd-anomaly in association with the number of major hospitals (**a**) and magnetic resonance imaging units (**b**) in the area

The Gd-anomaly showed strong positive correlations to the number of major hospitals (*r* = 0.88; *p* < 0.001) and their MRI units in the area (*r* = 0.89; *p* < 0.001) (Fig. 5).

## Discussion

This study investigated anthropogenic Gd in the Tone River, which runs through suburban areas of the Kanto region in Japan. All the samples displayed positive Gd anomalies with a zigzag distribution.

The current medical use of GBCAs for MRI is now commonly recognized as the origin of positive Gd-anomalies as first reported in 1996 [31]. Several studies have demonstrated that GBCAs are not removed in the dissolved phase in WWTPs [17, 32]. Our results demonstrated anthropogenic Gd contaminations in river water, with Gd-anomalies ranging from 121 to 6,545%, which can be related to discharges from WWTP outputs in the river.

Although there is a cluster of 6 major hospitals with a total of 13 MRI units near the basins from #6 to #7, the samples from these areas demonstrated the second and third lowest Gd-anomaly of the 15 sample points. The wastewater from these lesions is gathered into a WWTP downstream of them and discharged upstream of #8, meaning there is no discharge point in these areas. The water sample of #8 revealed increased anthropogenic Gd, and therefore, this result supports the hypothesis that anthropogenic Gd is not removed at WWTP. Similarly, there are 12 hospitals upstream of #11 to #12 and 18 hospitals upstream of #14, and water samples from these lesions demonstrated increased anthropogenic Gd concentrations. As shown in Fig. 5, there were strong correlations between the Gd-anomaly and the number of hospitals and their MRI units, suggesting that contrast-enhanced MRI performed in those hospitals are likely to be the cause of the anthropogenic Gd.

In our study, the degree of the Gd anomaly revealed a repeating decrease-and-increase trend—a zigzag distribution, but not a linear relationship. These variations can be made by balancing discharges from WWTP outputs, the confluence of tributaries, and deposition in sediments of the river. A previous study reported that anthropogenic Gd concentration in sediment was three orders of magnitude higher than in surface water of wetland, and sedimentation of Gd may occur through plant uptake and incorporation in organic biomass [33]. A similar mechanism is expected in river water and sediment. Although macrocyclic GBCAs are known to be highly stable, it has been reported that GBCAs used in clinical practice are susceptible to destruction by ultraviolet irradiation, regardless of their configurations [34]. Ultraviolet disinfection in WWTPs may be one of the causes of de-chelating. Furthermore, Gd^3+^ is known to combine with phosphate and form insoluble gadolinium phosphate [35, 36]. Phosphorus is essential for plant growth and is commonly used as a fertilizer in rice cultivation to increase yields. Excess nutrients that are not fully absorbed by crops run off the soil and cause phosphorus to accumulate in the water [37, 38]. As rice cultivation is widely practiced in Japan, water from paddy fields containing phosphate originating from fertilizers flows into rivers, which may be one of the causes of sedimentation.

Gd-anomaly dropped sharply at #15, which is 3 km from the estuary. The dilution of the seawater backflow may cause this. Although there is no WWTP upstream of #1 or major hospitals around it, the water sample from #1 displayed the third-highest Gd-anomaly of the 15 sample points. This may be due to oxidation ditches and septic tanks upstream of a tributary of the Tone River.

Compared with the previous report in 2000, the Gd-anomaly in Tone River increased from 851% (sampled in 1996) to 6,545%, *i.e.*, 7.7 times, over the past two decades, reflecting the increased use of GBCAs in hospitals [15]. Because the number of MRI examinations is still increasing and this trend will not be changing soon, it is expected that anthropogenic Gd concentration in the aquatic environment will also continue to increase in the future. Anthropogenic Gd compounds used as GBCAs are still stable in river water and remain in the aquatic environment for a longer period [39]. As a result, they can be eventually incorporated into aquatic organisms in the food chain and may be consumed by humans.

Sewage treatment technologies commonly used today can only remove about 10% of Gd, and the majority of anthropogenic Gd is discharged directly into rivers [40]. Reverse osmosis membrane technology is currently the only purification method that can remove almost all (99.85%) of the Gd [41]. Given the proportion of outpatients undergoing contrast-enhanced MRI scans, localized treatment of wastewater at individual hospitals may have limited effect and should therefore be used in WWTPs. However, the strategy of installing this equipment in all WWTPs is not economically practical at present, as it is very costly.

The simplest way to reduce the discharge of anthropogenic Gd into the aquatic environment would be to collect urine from the patient for at least 24 h after administration of the GBCA. For this purpose, urine should be collected at the patient’s home as well as in the hospital. In a trial conducted in Germany, there were 76% of participants who accepted integrating urine collection bags into their existing routines at future examinations with X-ray contrast agents [42]. As a potential method of retrieving anthropogenic Gd that has been discharged into rivers, a previous study reported the potential capability of water hyacinths in reducing anthropogenic Gd, but it is still not practical [43].

The most immediately feasible way to reduce anthropogenic Gd would be to use novel drugs providing the same diagnostic effect with half the dose of conventional GBCAs due to their high r1 relaxivity [11]. In any case, new public guidelines and the development of effective removal technologies are necessary to reduce future contamination of anthropogenic Gd.

While this study found evidence to support the theory of increased anthropogenic Gd in Japanese rivers, several limitations to this study should be considered.

First, the Tone River has the largest drainage area and the second-longest river length in Japan, and the sampling points are from 3 to 235 km from the estuary. Therefore, sample collections could not be completed in one day. However, as these two days were cloudy, there was no impact on the concentration of anthropogenic Gd due to rain dilution or dam releases, suggesting no influence on the results of the present study.

Second, this study did not examine the specific types of GBCAs used in each hospital or the frequency of their use. The concentration of Gd in effluent may be affected by the type of GBCA and the amount of its use. However, in Japan, linear GBCAs were already allowed to be used only when other GBCAs could not be used—banned in principle—by 2020. Therefore, we assume that the impact of variations in the type of GBCA on the results may be limited. Further studies including the frequency and amount of GBCA use in each hospital are desirable.

Third, our study was based on a single water sampling taken at 15 locations, lacking an estimate of the error. The selection of these specific sites may not have fully captured the variability in Gd concentrations across the river. Furthermore, annual dynamics may have been overlooked as the use of GBCAs and other anthropogenic activities may vary throughout the year. For more reliable results, more samples would need to be taken several times throughout the year over a more extensive area. Additionally, we did not take a sample from the headwaters. Further studies, including the spring water from the headwaters and multiple sampling, may be important.

In conclusion, our study demonstrated the updated situation of the REEs with a notable anomaly of Gd concentration in river water in Japan; the degree of Gd anomalies increased by 7.7 times over the past two decades. Because using GBCAs is essential in today’s clinical practice, the increasing use of GBCAs is inevitable, and the supply of anthropogenic Gd to the natural environment will continue. Therefore, more extensive investigations of anthropogenic Gd should be conducted in the future, such as the impact on living organisms, including bioconcentration and uptake into plants and risks to human health.

## Data Availability

All data used to support the findings of this study are included in the article.

## Abbreviations

GBCA: Gd-based contrast agent
Gd: Gadolinium
In: Indium
MRI: Magnetic resonance imaging
REE: Rare earth elements
WWTP: Wastewater treatment plant
